# Assessment and Implication of PAHs and Compound-Specific δ^13^C Compositions in a Dated Marine Sediment Core from Daya Bay, China

**DOI:** 10.3390/ijerph19084527

**Published:** 2022-04-09

**Authors:** Yan Lu, Dongmei Li, Xiaoyun Wang, Jianping Cao, Sheng Huang, Peng Zhou

**Affiliations:** 1 Guangdong University of Petrochemical Technology, Maoming 525000, China; hyss.yanlu@163.com (Y.L.); caojp_iso@163.com (J.C.); 2 South China Sea Environment Monitoring Center, State Oceanic Administration (SOA), Guangzhou 510300, China; lidmay@foxmail.com (D.L.); hsheng1984@163.com (S.H.); 3 Nansha Islands Coral Reef Ecosystem National Observation and Research Station, Guangzhou 510300, China; 4 Key Laboratory of Marine Environmental Survey Technology and Application, Ministry of Natural Resources (MNR), Guangzhou 510300, China; 5 Sixth Geological Brigade of Hubei Geological Bureau, Xiaogan 432000, China; wxykrdm@gmail.com

**Keywords:** polycyclic aromatic hydrocarbons (PAHs), compound-specific carbon stable isotope, marine sediment, sources identification, Daya Bay

## Abstract

PAHs in a sediment core covering ~120 years from Daya Bay in South China Sea were extracted using Soxhlet and high performance thin layer chromatography, and the compound-specific δ^13^C were analyzed using gas chromatography–combustion–isotopic ratio mass spectrometry. The concentrations of PAHs ranged from 99.3 to 676 ng g^−1^, with high molecular weight PAHs as a key component. PAHs’ compound-specific δ^13^C ranged from −35.02‰ to −16.14‰. The patterns of 16 PAHs, molecular ratios, and compound specific δ^13^C compositions indicate important pyrolytic and petrogenic sources: PAHs derived predominantly from pyrogenic sources (including coal and wood incomplete combustion) before the 1960s, while after the 1960s, they derived predominantly from mixed pyrogenic and petrogenic sources (including automotive exhaust emissions, oil spills, and coal and wood incomplete combustion). Our results can provide important insights into organic pollution emissions influenced by human activities and the urbanization of Daya Bay.

## 1. Introduction

Polycyclic aromatic hydrocarbons (i.e., PAHs) are a class of compounds containing two or more fused benzene rings, and are ubiquitous in atmosphere, soil, waterways, and oceans, as well as in the food chain [1]. PAHs are identified as priority pollutions by the U.S. Environmental Protection Agency owing to their hydrophobicity, lipophilicity, toxicity, and genotoxicity [2,3,4]. PAHs are derived from three major sources: petrogenic—derived from slow maturation of organic matter under geothermal gradient conditions; pyrogenic—derived from incomplete combustion of organic matter; and short-term diagenetic products—derived from biogenic precursors [1,5,6,7,8,9]. Both the molecular fingerprints (or molecular indices) method and the compound-specific δ^13^C isotopic fingerprint technique have been successfully used in the source identification and apportionment of PAHs pollution, as well as their biological effects [10,11,12]. The molecular fingerprint investigation is used often, but such an approach suffers from many complications [13,14,15,16,17]. The compound-specific δ^13^C isotopic technique using gas chromatography-stable carbon isotope ratio mass spectrometry (GC-IRMS) has proven since the early 1990s to be a useful tool to investigate the carbon cycle at the molecular level [6,18,19,20,21,22,23]. PAHs have source-specific isotope values and patterns [19,20], thus specific carbon isotope analysis of PAHs is a useful tool for source identification and apportionment, in order to further understand the biogeochemical process [5,20,24,25,26,27,28,29].

Sediments might provide useful information to understand the cycling of toxic chemicals and to allow assessment of the effectiveness of environmental legislations [2]. In the present study, a method for the determination of the compound-specific δ^13^C of PAHs was developed for Soxhlet extraction, high performance thin layer chromatography (HPTLC), and GC-IRMS. We have measured 16 PAHs’ composition and isotopic compositions in a 60 cm long sediment core from Daya Bay. Historical records of 16 PAHs’ composition, specific molecular ratios, and compound specific δ^13^C compositions were analyzed to determine the trend and extent of organic pollution accumulation, and to reconstruct the shift of contributing combustion sources. These results are also expected to improve our knowledge on temporal trends, congeries, and the distribution of PAHs, as well as contribute to understanding the relative importance of the potential sources. Furthermore, our study can provide a background estimation of baseline data for future responses of the marine environment to anthropogenic activities in the Hong Kong–Zhuhai–Macao Greater Bay Area.

## 2. Materials and Methods

### 2.1. Study Area and Sampling Strategy

Semiclosed Daya Bay was one of the main aquaculture areas at the Guangdong–Hong Kong–Macao Greater Bay Area in the Pearl River Estuary owing to its excellent water quality and rich biological ecosystem in the past [30,31,32,33,34,35,36]. However, it has been severely impacted by human activities since the 1980s, owing to a rapid expansion of aquacultural, industrial, and agricultural activities, e.g., petrochemical, plastic, printing, and other industries; the nuclear power plant base (also named Daya Bay DNPP base); and the commercial shipping harbor [37,38,39]. There is strong evidence, presented previously, of a common petroleum contamination source in Daya Bay [37]. Some studies have been made on the concentrations of PAHs in sea water and sediment [40,41,42,43,44,45,46,47], but there appear to be few studies on the isotopic compositions of the PAHs.

**Figure 1:**
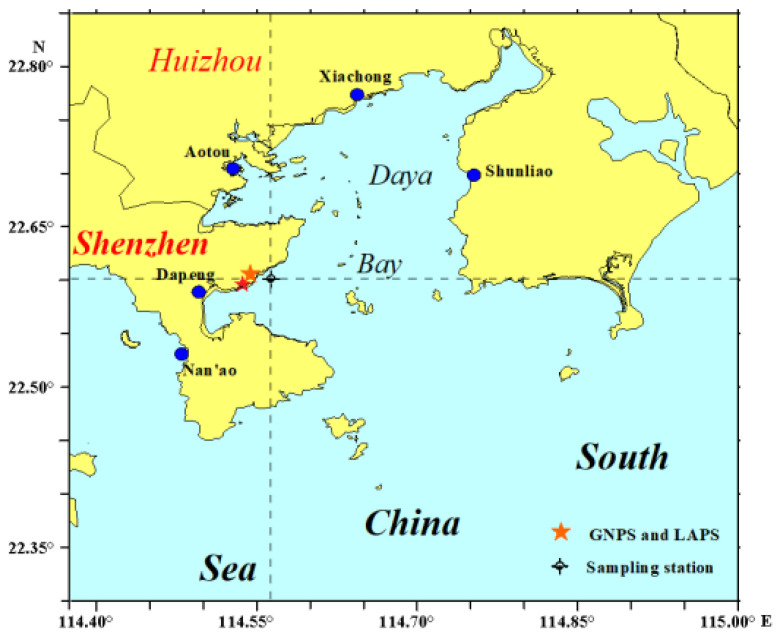
Sampling station of the sediment core from Daya Bay.

A 60 cm long sediment core was collected from the western area of Daya Bay on 28 July 2010, as seen in Figure 1. The core was sliced into 30 subsamples with 2.0 cm thin sections, air-dried, ground, and screened (200 mesh). The ^210^Pb and ^226^Ra were analyzed using a HPGe γ-spectrometer (Canberra Industries Inc., Meriden, CT, USA), with a broad energy detector (Model BE5030) and a lead shield (Model 747) The sediment core with a period of about 120 years (from 1891 to 2010) was dated using the ^210^Pbex method of the constant flux constant sedimentation model (CF or CFCS) [48,49]. PAHs and compound-specific δ^13^C isotopic compositions were analyzed at Guangdong University of Petrochemical Technology. PAHs in sediment were extracted using Soxhlet high performance thin layer chromatography (HPTLC), and then compound-specific carbon isotope and molecular composition were analyzed by gas chromatography–combustion–isotope ratio mass spectrometry (GC-C-IRMS).

### 2.2. Treatment and Separation Procedures

**Figure 2:**
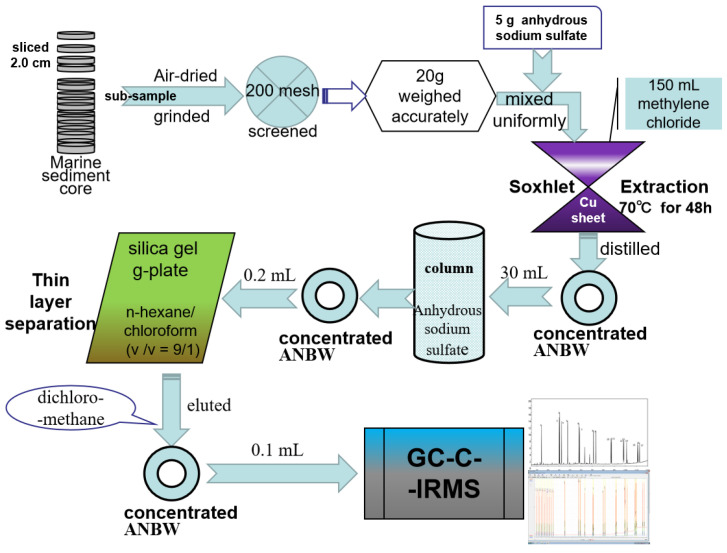
The flow chart of treatment and separation procedures of PAHs and compound-specific carbon stable isotope in marine sediment.

Figure 2 shows the flow chart of treatment and separation procedures of PAHs and compound-specific carbon stable isotope in marine sediment. Total PAHs (∑PAHs) is the sum of the 16 USEPA priority congeners: naphthalene (Nph), acenaphthylene (Acy), acenaphthene (Acp), fluorene (Flr), phenanthrene (Phe), anthracene (Ant), fluoranthene (Fluo), pyrene (Pyr), benzo(a)anthracene (BaA), chrysene (Chr), benzo(b)fluoranthene (BbF), benzo(k)fluoranthene (BkF), benzo(a)pyrene (BaP), indeno(1,2,3-cd) pyrene (InP), dibenzo(a,h)anthracene (DhA), and benzo(g,h,i)perylene (BgP).

#### 2.2.1. Extraction of PAHs

The sediment sub-samples were air-dried in a clean dish under the cool and ventilated conditions, then ground and screened through a 200-mesh screen before Soxhlet extraction. PAHs were extracted using Soxhlet followed by HPTLC, and the molecular composition and compound-specific δ^13^C isotope were then analyzed by GC-C-IRMS [50,51]. In brief, 20 g of subsample with 5 g of anhydrous sodium sulfate in a glass filter paper tube was placed into a distillation bottle (250 mL). After adding 150 mL of methylene chloride and several copper sheets (to remove organic sulfide), Soxhlet extraction was achieved under a temperature of 70 °C for 48 h [52]. Extract was collected, concentrated to ~30 mL by rotary distillation, passed through a column of anhydrous sodium sulfate, and re-concentrated to 0.2 mL using an automatic nitrogen blower (Model XT-NS1, Shanghai Xintuo, Shanghai, China). Thin-layer separation on silica gel g-plate was performed using the extender of n-hexane/chloroform (*v*/*v* = 9/1).

#### 2.2.2. Determination of GC-C-IRMS

**Figure 3:**
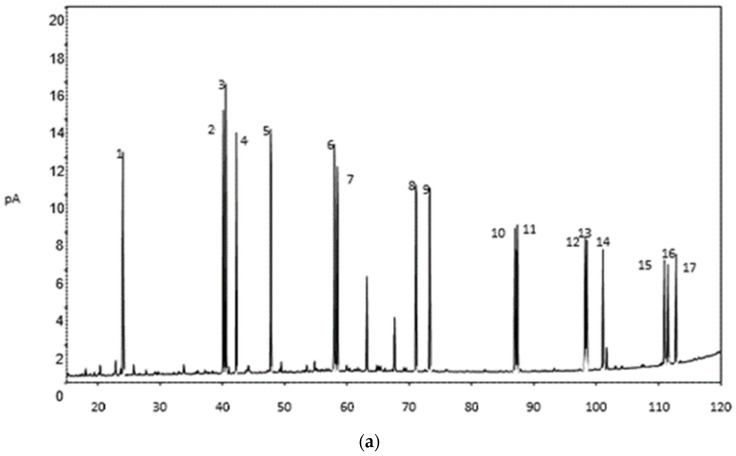

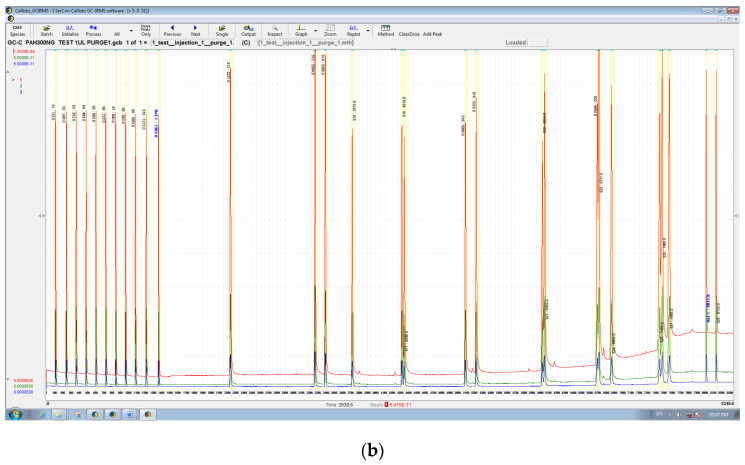
Peaks of PAHs’ hybrid standard gas chromatogram using GC and GC-C-IRMS. (**a**) Peaks of PAHs using GC: 1-Nph, 2-Acy, 3-Six methyl benzene, 4-Acp, 5-Flr, 6-Phe, 7-Ant, 8-Fluo, 9-Pyr, 10-BaA, 11-Chr, 12-BbF, 13-BkF, 14-BaP, 15-LnP, 16-DhA, and 17-Bgp; (**b**) peaks of 16 PAHs using GC-C-IRMS.

A method for the compound-specific carbon stable isotope analysis of 16 PAHs in marine sediments by GC-C-IRMS was established. The PAHs’ component color bands were divided under ultraviolet lamp, eluted with dichloromethane, collected, and concentrated to ~0.1 mL to analyze by GC-C-IRMS (IRMS, Model Sercon 20-22, Sercon Ltd., Enfield, UK; GC-CP joint, Sercon Ltd., Crewe, UK; Gas chromatograph, Model 7890B, Agilent Technologies Co., Ltd., Santa Clara, CA, USA). The determination details are as follows: the sample injection volume was 1 μL using full-automatic stream injector in splitless mode; HP-5 quartz capillary column (30 m × 0.32 mm × 0.25 μm); vaporizing chamber temperature of 300 °C; temperature condition, initial 40 °C (holding time for 4 min), rise by 2 °C/min to 290 °C for 10 min (holding time for 10 min); the flow rate of helium gas carrier was 1 mL/min; and the oxidation furnace temperature was 860 °C. The IRMS conditions were electron bombardment (EI) ion source, acceleration voltage of HT 2117 V, well voltage trap of 600 V, and lamp current emission of 1580 μA. Figure 3 shows the peaks of PAHs’ hybrid standard gas chromatogram using GC and GC-C-IRMS.

### 2.3. Quality Control

All data are subject to quality control procedures (including blanks, the Standard Reference Materials, and spiked samples), which are detailed by [50,51]. In brief, based on five analyses of the same Standard Reference Material of 16 PAHs (2000 ng/mL for the single homologue) (the Accu Standard Inc., New Haven, CT, USA), the RSDs (relative standard deviations, *n* = 5) were 0.67–9.00% for the single homologues. Estimated recoveries for each analyte were 72.8~112.3% for 16 PAHs, when one spiked sample was measured for every 10 samples in the actual operation. The LD (limit of detection) for 16 PAHs ranged from 1.11 ng (Acy) to 6.41 ng (Chr), and procedural blanks were always below the LD.

For repeatability of compound-specific δ^13^C isotopic compositions, GC-C-IRMS was investigated using the isotopic standard reference substances and gas calibration of CO_2_ cylinders, as described in detail by Lu et al. (2018) [50]. Standard Reference Materials of δ^13^C isotope were adopted from IAEA-C8 (Oxalic Acid, δ^13^CV-PDB = −18.3 ± 0.2‰) (International Atomic Energy Agency, Vienna, Austria) and EMA-P2 (δ^13^CV-PDB = −28.19 ± 0.14‰) (IRMS certified reference material, NB/13257, Elemental Microanalysis Ltd, UK). References of carbon dioxide gas (CO_2_, >99.999%), helium gas carrier (>99.999%), oxygen (>99.999%), high-purity hydrogen gas (>99.999%), and dry air were used in the procedures. The δ^13^C-RSD of Standard Reference Materials was less than −0.03‰, and the RSD of reference CO_2_ cylinder gas was only 0.11‰.

## 3. Results and Discussion

All data are from the concentrations of dry sediment (ng g^−^^1^_dryweight_) (as seen in Table S1). The concentrations of total PAHs (i.e., ∑PAHs) were 99.3–677 ng g^−^^1^ with the mean values of 304 ng g^−^^1^. PAHs may be divided into low molecular weight PAHs (LPAHs, including Nph, Acy, Acp, Flr, Phe, and Ant) and high molecular weight PAHs (HPAHs, including Fluo, Pyr, BaA, Chr, BbF, BkF, BaP, InP, DhA, and BgP). The concentrations of LPAHs and HPAHs were 7.4–252 and 45.4–477 ng g^−^^1^, respectively, with mean values of 80.6 and 223 ng g^−^^1^, respectively. Here, the PAHs in the sediment in Daya Bay and its adjacent areas are shown in Table 1. The ∑PAHs were at a similar level to those in Dapeng’ao bay, as well as those reported in Daya Bay [44], in the Pearl River Estuary [51], and in the northern South China Sea [52]. However, they are less than those in Dapeng Bay [42], in Shenzhen nearshore [43] (Tang et al., 2017s), and in the middle area of the South China Sea [53]. Therefore, the PAHs’ contamination in our study was at a lower medium level in Daya Bay [41,42,46,54].

The LPAHs with 2–3 benzene rings are mainly some volatile aromatic hydrocarbons (naphthalene, fluorene, phenanthrene, and anthracene), which are toxic to aquatic organisms. The HPAHs with 4–6 benzene rings are mainly some high-boiling and non-volatile aromatic hydrocarbons (pyrene, fluoranthracene, xanthene, benzo (a) pyrene, and halobenzene), which have carcinogenic, teratogenic, and mutagenic effects [1,57,58]. The concentrations of the HPAHs were higher than those of LPAHs. The LPAHs were derived mainly from the 3-rings PAHs contributions (3.31–68.2%, with the mean value of 28.4%). The mean percentages of the 4-rings, 5-rings, and 6-rings contributions to HPAHs were 46.70% (7.82–90.7%), 24.02% (0.00–82.9%), and 29.30% (0.00–83.2%), respectively. The mean percentages of 4-rings, 5-rings, and 6-rings contributions to ∑PAHs were 31.6% (6.00–72.3%), 18.1% (0.00–80.2%), and 21.7% (0.00–63.9%), respectively, suggesting that the PAHs were dominated by the HPAHs with 4–6 benzene rings.

**Figure 4:**
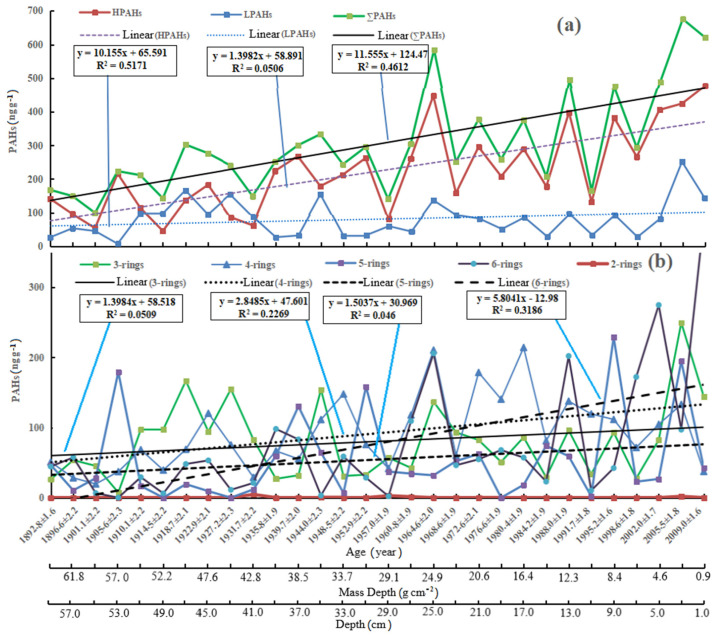
Profiles of PAHs varied with age (depth and mass depth) in marine sediment core from Daya Bay. (**a**) The concentrations of ∑PAHs, HPAHs, and LPAHs with age; (**b**) the concentrations of the 3-rings, 4-rings, 5-rings, and 6-rings PAHs with age.

Figure 4 shows that profiles of PAHs varied with age in the sediment core. The concentrations of ∑PAHs, HPAHs, and LPAHs have some positive correlations with age, as seen in Figure 4a. Although the positive correlation between LPAHs and ages is relatively weak (y = 1.3982x + 58.891, R^2^ = 0.0506), the positive correlations are very significant between the ∑PAHs’ and HPAHs’ concentrations and ages (∑PAHs: y = 11.555x + 124.47, R^2^ = 0.4612; HPAHs: y = 10.155x + 65.591, R^2^ = 0.5171). The significant- increase in the concentrations of ∑PAHs with age is consistent with those surveyed by Chi et al. (2005) [40]. For the slope of the fitted straight line, the results show that the annual growth rates of the concentrations of ∑PAHs and HPAHs are much greater than that of LPAHs. Similarly, the concentrations of 3-rings, 4-rings, 5-rings, and 6-rings PAHs have some positive correlations with ages, as seen in Figure 4b. The positive correlations between the 4-rings (y = 2.8485x + 47.601, R^2^ = 0.2269) and 6-rings PAHs (y = 5.8041x − 12.98, R^2^ = 0.3186) and ages are higher than those of the 3-rings and 5-rings PAHs. For the slope of the fitted straight line, the results show that the annual growth rate of the six-rings PAHs is the largest, followed by the 4-rings, 5-rings, and 3-rings PAHs.

### 3.1. General Trends of PAHs in Sediment Core

At the depths of 0–4 cm, the concentrations of ∑PAHs with 621 (2009.0 ± 1.6 a) and 677 ng g^−^^1^ (2005.5 ± 1.8 a) were higher than those of other layers. Additionally, two high peaks (475 and 585 ng g^−^^1^) occurred at the depth of 8–10 cm (1995.2 ± 1.6 a) and 24–26 cm (1964 ± 2.0 a). Three peak values of HPAHs occurred at the depths of 0–2 cm, 2–4 cm, and 24–26 cm (1964.6 ± 2.0 a), and the highest peak of 252 ng g^−^^1^ for LPAHs occurred at the depth of 2–4 cm (2005.5 ± 1.8 a). The high peak at the depth of 8–10 cm is due to the higher values of the 5-rings BaP (103 ng g^−^^1^), 4-rings BbF (120 ng g^−^^1^), and 3-rings Phe (79.2 ng g^−^^1^). These high values may be related to the large-scale development of Daya Bay in the 1990s. In January 1994, the Sino foreign joint Daya Bay Huade Petrochemical Co., Ltd. was established. Then, the magnificent construction of Mabianzhou island was opened with the “first explosion in Daya Bay” in September 1994, when about 1024 tons of explosives filled 920,000 cubic meters of rock in the sea. Thus, the marine engineering development and subsequent operation of petrochemical companies will lead to more high molecular weight PAHs entering the atmosphere, water, and sediment of Daya Bay than before. Another high peak occurred at the depth of 24–26 cm owing to the higher values of the 6-rings BgP (142 ng g^−^^1^), 4-rings BaA (150 ng g^−^^1^), and 2-rings Acy (107 ng g^−^^1^). The concentration of the 2-rings Acy (107 ng g^−^^1^) in thirty sub-samples is the highest because it came from grass, wood, and coal combustion at the period of material poverty and economic backwardness. The high values of both the 6-rings BgP (142 ng g^−^^1^) and 4-rings BaA (150 ng g^−^^1^) may be caused by other reasons to be explored in future.

Combined with the vertical distribution of radionuclides in a marine sediment core, [48], grain-size, water content, TOC, TIC, TC, LOI (loss on ignition), TN, BSi, and TP [49], the sediment process can be divided into four periods based on the PAHs’ profiles: 1980.4–2009.0, 1960.8–1976.6, 1931.7–1960.8, and 1892.8–1927.2. In the four periods, the concentration ranges of ∑PAHs were 165.1–677, 251.4–585, 141–334, and 99.2–303 ng g^−^^1^, with their mean values of 422, 356, 252, and 202 ng g^−^^1^, respectively. The increasing trend of the concentrations of ∑PAHs with age was significant.

For the LPAHs in the four periods, the concentration ranges were 29.0–252, 51.1–137, 26.9–155, and 7.4–167 ng g^−^^1^, with the mean values of 93.6, 90.9, 58.5, and 82.5 ng g^−^^1^, respectively. There is no an increasing trend in the concentrations of LPAHs with age. For the HPAHs in the four periods, the concentration ranges were 132–477, 158–448, 61.6–218, and 45.4–216 ng g^−^^1^, with the mean values of 328, 278, 194, and 119 ng g^−^^1^, respectively. Similar to the ∑PAHs, the increasing trend of the concentrations of HPAHs with age was significant. The concentrations of HPAHs and ∑PAHs increased significantly (the linear correlation coefficient, R^2^ = 0.88), whereas the concentrations of LPAHs increased slightly. Additionally, the increasing trend in the concentrations of ∑PAHs with age after the 1960s was significantly greater than that before the 1960s.

In the four periods above, the percentages of LPAHs to ∑PAHs were 40.8% (3.31–68.2%), 25.9% (10.7–59.7%), 25.5% (19.7–37.0%), and 20.3% (9.36–37.2%), respectively. The percentages of HPAHs to ∑PAHs were 59.2% (31.8–96.7%), 74.1% (41.3–89.3%), 74.5% (62.9–80.3%), and 79.7% (62.8–90.6%). The profiles of ∑PAHs, LPAHs, and HPAHs suggested the history of the PAHs input to Daya Bay. The increase in the concentrations of ∑PAHs accelerated significantly before and after the 1960s, and the percentages of the LPAHs and/or HPAHs to the ∑PAHs changed significantly before and after the 1930s. After the 1930s, PAHs in the sediments of Daya Bay were derived mainly from incomplete combustion of grass, wood, and coal. Furthermore, accumulation of PAHs accelerated after the 1960s, because, before 1960 Daya Bay, was dominated mainly by slow and primitive agriculture. Since the early 1960s, the urban construction and industry around Daya Bay had begun to develop rapidly, especially since the 1980s. Petrochemical, plastic, printing, and other industries, as well as harbors, have been built around the sea area. Massive economic growth and urban development in the region have led to excessive release of waste into the bay [33,35,59,60].

### 3.2. Molecular Ratios of Specific Aromatic Compounds and Possible Sources

**Figure 5:**
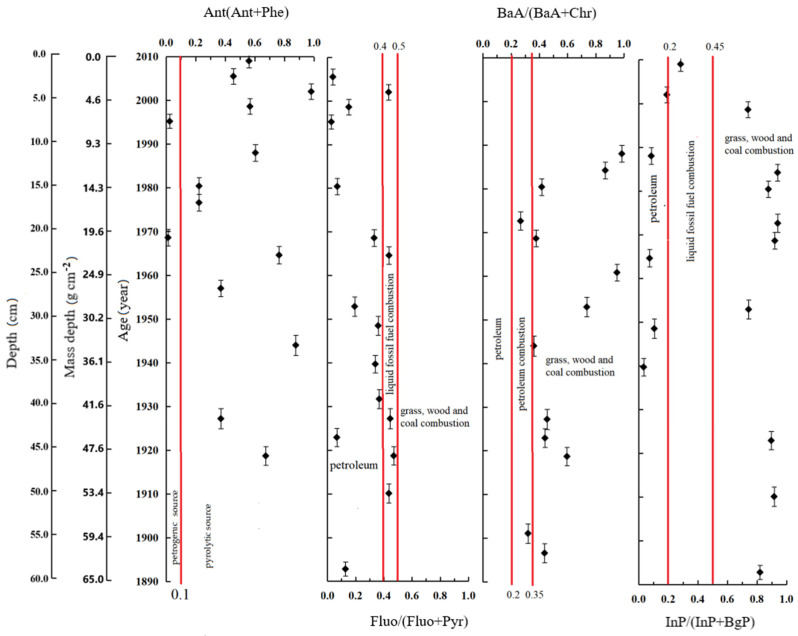
Molecular ratios of specific aromatic compounds of PAHs with age (depth and mass depth) and their potential sources.

As shown in Table 2, molecular indices (fingerprints) based on the concentration ratios of selected PAHs are used to extensively identify the PAHs from petrogenic and pyrogenic origins, as well as to distinguish source apportionment [61,62,63,64,65,66,67,68,69,70,71,72]. To distinguish between petrogenic and pyrolytic sources, Figure 5 shows the isomer ratios of Ant/(Ant + Phe), Fluo/(Fluo + Pry), InP/(InP + BaP), and BaA/(BaA + Chr), as seen in Table S2.

The ratios of Ant/(Ant + Phe) ranged from 0.03 to 0.98 (the mean value of 0.48), suggesting that the PAHs inputs came largely from pyrolytic sources [64,69,72,73]. The ratios of InP/(InP + BaP) ranged from 0.03 to 0.94; before 1931.7(±2.2a), the ratios of InP/(InP + BaP) were higher than 0.5, suggesting that the PAHs inputs came from the combustion of grass, wood, and coal; during 1935–2000, the ratios of InP/(InP + BaP) varied greatly between 0.03 and 0.94, suggesting that the PAHs inputs came from both petrogenic and pyrolytic sources; after the 2000s, the ratios (0.20–0.05) suggested that the PAHs inputs came from liquid fossil fuel combustion [72]. Before the 1900s, the ratios of BaA/(BaA + Chr) varied greatly from 0.27 to 0.99 (the mean value of 0.55), suggesting that the PAHs inputs came from pyrolytic sources and some petrogenic sources [72]. However, the ratios of Fluo/(Fluo + Pry) ranged from 0.13 to 0.47 (mean value of 0.27), suggesting that the PAH inputs should have come from a petroleum source [68,72,73]. This assessment is not in agreement with those based on the ratios of InP/(InP + BaP) and BaA/(BaA + Chr).

### 3.3. Molecular and Isotopic Compositions (Compound-Specific δ^13^C Compositions)

The mean values of compound-specific δ^13^C ( δ^13^CV-PDB) of LPAHs, HPAHs, and ∑PAHs in the sediment core were −22.72‰ (−29.47–−20.71‰), −24.46‰ (−26.80–−20.86‰), and −23.90‰ (−35.02–−16.14‰), respectively, as seen in Tables S3 and S4. The mean δ^13^C isotopic composition in HPAHs was heavier than that in LPAHs. The compound-specific δ^13^C of PAHs was affected by multiple potential sources, e.g., plants and fossil fuels, wood combustion, the microbial degradation of hydrocarbon, gasoline and diesel exhaust emissions, and so on [7,8,15,16,23,26,27,29,77,78,79,80,81].

**Figure 6:**
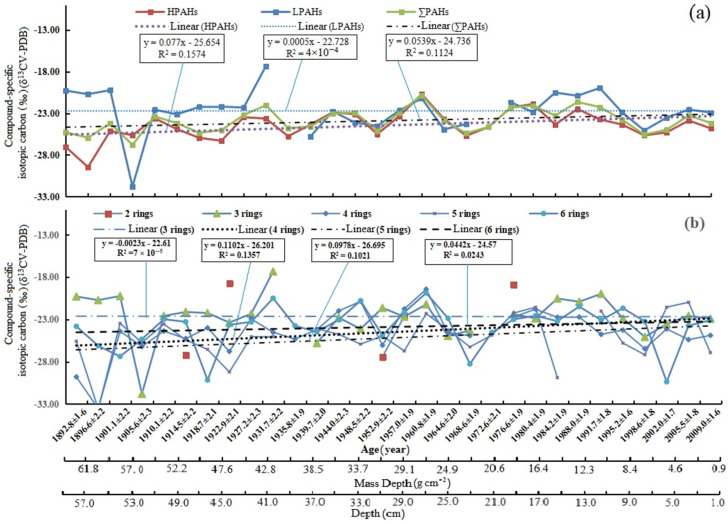
The profiles of the mean δ^13^C values of (**a**) LPAHs, HPAHs, and ∑PAHs, and (**b**) 2-rings, 3-rings, 4-rings, 5-rings, and 6-rings.

**Figure 7:**
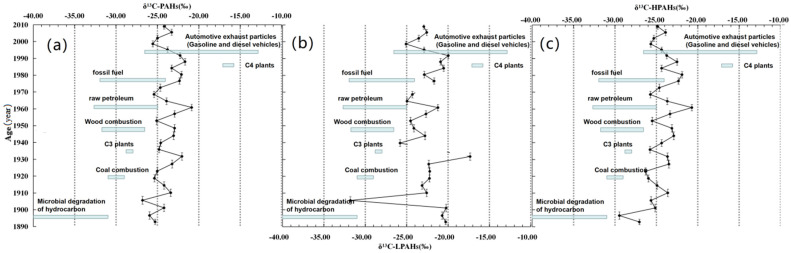
The profiles of the mean δ^13^C values of LPAHs, HPAHs, and ∑PAHs and their potential sources. (**a**) the mean δ^13^C values of ∑PAHs; (**b**) the mean δ^13^C values of LPAHs; (**c**) the mean δ^13^C values of HPAHs. Note: The eight potential sources are as follows: (1) C3 plants combustion (approximately from −28.80‰ to −28.00‰) [8]; (2) C4 plants combustion (from −17.10‰ to −15.80‰) [8]; (3) automotive exhaust particles (gasoline/diesel vehicles) (from −12.90‰ to −26.00‰) [28,29,78]; (4) wood combustion (from −31.60‰ to −26.80‰) [26,77]; (5) Coal combustion (from −31.00‰ to −29.00‰) [23,29]; (6) fossil fuel (from −32.00‰ to −24.00‰) [26]; (7) crude oil/petroleum (−33.70‰ to −25.40‰) [80,81]; and (8) microbial degradation of hydrocarbon (from −62.00‰ to −31.00‰) [15,16]).

The profiles of the mean δ^13^C values of LPAHs, HPAHs, and ∑PAHs and their potential sources are shown in Figure 6 and Figure 7. The mean compound-specific δ^13^C of 16 PAHs became negative with depth (with age). There is not a positive correlation between LPAHs (y = 0.0005x − 22.728, R^2^ = 4 × 10^−6^) or the 3-rings PAHs (y = 0.0023x − 22.61, R^2^ = 7 × 10^−^^5^) and ages, suggesting that the LPAHs are not necessarily related to age. However, there is a weak positive correlation between HPAHs (y = 0.077x − 25.654, R^2^ = 0.1574) and ages, as well as between ∑PAHs (y = 0.0539x − 24.736, R^2^ = 0.1124) and ages. The trend indicates that the source of PAHs increased with age. Similarly, as seen in Figure 6, there is a weak positive correlation between the 4-rings PAHs (y = 0.1102x − 26.201, R^2^ = 0.1357) and age, as well as between the 5-rings PAHs (y = 0.0978x − 26.695, R^2^ = 0.1021) and age. This suggests that the contribution of the 4-rings and 5-rings PAHs is great. In Figure 7, the end number of PAHs from automotive exhaust particles (gasoline/diesel vehicles) is more positive than other sources. Thus, our results show that the contribution of the PAHs sources from automotive exhaust particles increased with age.

As seen in Figure 6 and Figure 7, the δ^13^C values before the 1960s were more negative than those after the 1960s. This result suggests that the PAHs mainly came from the incomplete combustion of wood and coal before the 1960s. This is because the area around Daya Bay remained at an agrarian age with underdeveloped industry, and the social economy came mainly from simple farming, fishing, and other low productivity activities. After the 1960s, the δ^13^C compositions became heavier with age, and a peak value (−25.86‰) occurred in 1960. The PAHs could have been introduced not only from the incomplete combustion of wood and coal, but also from automotive exhaust particles (including gasoline/diesel vehicles and ships as well as oil spills at sea). Following the land reform around Daya Bay carried out in the late 1950s and early 1960s, the social productivity was liberated and human activities were intensified. Moreover, some high-efficiency and mechanical activities observed, e.g., gasoline/diesel vehicles and large tonnage mechanical/electrical ships gradually replaced the old and manual activities observed during the farming/fishing period. Especially in the late 1980s and early 1990s, a large number of industries such as wharves and petrochemical factories, especially thermal power plants and petrochemical refineries, were established and began to operate [82,83,84]. These human activities accelerated the shift of PAHs sources. Automotive exhaust particles then became the main source of PAHs.

## 4. Conclusions

The historical records of the compositions, specific molecular ratios, and compound specific δ^13^C of 16 PAHs are analyzed to determine the trend and extent of organic pollution accumulation. The profiles of PAHs’ compositions in the sediment core reflect the impact of human activities on the natural ecological environment. The deposition of PAHs affected by anthropogenic disturbances over Daya Bay in the past 40+ years was presented. The specific molecular (isomer) ratios of PAHs indicated that PAHs before the 1960s came mainly from combustion sources, while PAHs after the 1960s came from a mixed source of combustion and petroleum owing to automobile and shipping exhaust along with leakage. The compound-specific δ^13^C of PAHs suggested that PAHs in the earlier period (before the 1960s) were generated mainly from the incomplete combustion of wood, coal, and so on. In the later period (after the 1960s), PAHs are thought to result from not only the incomplete combustion of wood and coal, but also the exhaust emissions from automotive vehicles and shipping, as well as oil spills at sea. Additionally, our results are useful for understanding the magnitudes and historical variations of organic pollution along the coasts of the South China Sea. In particular, it is essential to better understand the emissions of organic pollutions and protect the marine ecological environment in the Hong Kong–Zhuhai–Macao Greater Bay Area.

## Figures and Tables

**Table 1:** The PAHs in the sediments in Daya Bay and its adjacent areas (ng g^−^^1^).

Sea Area	Rang	Mean	Reference
Sediment core W2(2) from Daya Bay	99.3~676.5	303.6	In the study
Sediment from Daya Bay	115~1134	-	[41,46,47]
Surfance sediment from Daya Bay	42.5~158.2	126.2	[45]
Surfance sediment in Dapeng’ao bay	237.3~1139	-	[44]
	256.7~744.1	-	
Sediment from Daya Bay	140~491	310 ± 92.4	[55]
Sediment from Dapeng Bay	216.6~1314	572.5	[42]
Sediment core from Daya Bay	77.0~306.0	192.0	[40,45]
Sediments of Shenzhen nearshore including Daya Bay	227.5~3897	870. 6	[43]
Sediment core No. 10 from Daya Bay	118.1~319.9	210.2	[45]
Sediment from the Lingdingyang of the Pearl River estuary	143.9~522.7	287.05	[53]
Sediment from thePearl River Estuary	144.0~1289	430 ± 216	[55]
	126.1~3829	563.5	[54]
Sediment from the northern South China sea	274~335	304	[55]
Sediment from the middle area in South China Sea	276.4~792.2	430.6	[56]

**Table 2:** Molecular ratios of specific aromatic compounds and their possible sources of PAHs.

Molecular Ratios		Phe/Ant	Antt/(Ant + Phe)	Fluo/(Fluo + Pyr)	BgP/InP	InP/(InP + BaP)	BaA/Chr	BaA/(BaA + Chr)
possible sources		>10 or 15 petrogenic source	<0.10 petrogenic source	<0.50 petroleum	Ratio >1 is relatively high in automotive exhaust particles	<0.20 petroleum	≤0.40 Petrogenic origin	<0.20 petroleum
				0.4–0.5 * liquid fossil fuel combustion		0.2–0.5 * liquid fossil fuel combustion		0.2–0.35 ** petroleum combustion
		<10 pyrolytic sources	>0.10 pyrolytic source	>0.50 grass, wood, and coal combustion		>0.50 grass, wood, and coal combustion	>0.90 pyrolytic origin	>0.35 grass, wood, and coal combustion
our study	Mean Range ^#^	9.19 0.02–74.4	0.48 0.01–0.98	0.27 0.13–0.47	4.57 0.06–28.6	0.57 0.03–0.94	7.96 0.36–69.1	0.55 0.27–0.99
reference		[64,69,72,73]		[68,72,73]	[74,75]	[72]	[64,76]	[71,72]

Notes: ^#^ The data below the limit of detection are ignored when calculating their mean values and ranges. * Combustion from liquid fossil fuel including vehicle and crude oil; ** petroleum combustion, especially liquid fossil fuel, vehicle, and rude oil combustion.

## Data Availability

The data presented in this study are available in the insert article here.

## Supplementary Materials

The following supporting information can be downloaded at https://www.mdpi.com/article/10.3390/ijerph19084527/s1, Table S1. The concentrations of PAHs (ng g^−^^1^) in marine sediment core from the Daya Bay. Table S2. Molecular ratios of specific aromatic compounds in the marine sediment core from Daya Bay. Table S3. Compound-specific δ^13^C PDB(‰) isotopic compositions of PAHs in the marine sediment core from Daya Bay. Table S4. Summary of δ^13^C PDB(‰) isotopic compositions of PAHs in marine sediment core from Daya Bay.
