# Supplementary material for: Assessment and Implication of PAHs and Compound-Specific δ13C Compositions in a Dated Marine Sediment Core from Daya Bay, China
Source: Int J Environ Res Public Health. 2022 Apr 9;19(8):4527. doi: 10.3390/ijerph19084527 (PMC9029777; doi:10.3390/ijerph19084527)
Supplement: Supplementary file 1 [file ijerph-19-04527-s001.zip › ijerph-1632840-supplementary.pdf]

Supplementary Materials

Table S1. The concentrations of PAHs (ng g⁻¹) in the marine sediment core from Daya Bay.

Depth (cm)	Mass depth (gcm^{-2})	Calendar	PAHs (ng/g) 2-rings	3-rings						4-rings					5-rings				6-rings				LPAHs	HPAHs	∑PAHs
			Nph	Acy	Acp	Flr	Phe	Ant	M3	Fluo	Pyr	BaA	Chr	M4	BbF	BkF	BaP	M5	InP	DhA	BgP	M6			
0–2	0.9	2009.0±1.6	LD	LD	LD	13.0	57.6	73.6	144	LD	LD	37.3	LD	37.3	22.9	2.3	17.4	42.6	104	28.8	264	397	144	477	621
2–4	2.8	2005.5±1.8	1.9	LD	98.7	LD	82.2	68.8	250	3.1	76.7	LD	53.2	133	2.7	18.9	173	195	LD	79.9	17.1	97.0	252	425	677
4–6	4.6	2002.0±1.7	LD	0.1	0.5	8.6	1.3	71.7	82.2	18.8	24.5	61.2	LD	105	23.9	LD	2.8	26.7	49.9	13.0	212	275	82.1	406	488
6–8	6.5	1998.6±1.8	LD	LD	LD	5.3	9.6	12.6	27.5	8.3	46.4	16.8	LD	71.5	4.7	8.9	9.2	22.8	10.8	158	3.8	172.2	27.5	266	294
8–10	8.4	1995.2±1.6	LD	LD	11.2	0.5	79.2	2.0	92.9	1.4	48.8	LD	61.3	112	120	6.1	103	229	LD	35.6	6.6	42.2	92.8	383	476
10–12	10.2	1991.7±1.8	LD	LD	31.4	2.0	LD	LD	33.4	LD	119	LD	LD	120	LD	2.1	LD	2.1	5.2	5.0	LD	10.2	33.4	132	165
12–14	12.3	1988.0±1.9	LD	LD	41.2	33.0	8.7	13.3	96.2	LD	82.3	54.5	0.8	138	12.1	24.7	22.4	59.2	13.3	46.2	143	202	96.2	399	495
14–16	14.3	1984.2±1.9	LD	LD	LD	1.7	LD	27.4	29.1	LD	35.5	39.5	6.0	81	7.1	9.9	57.0	74	17.2	4.6	1.1	22.9	29.0	178	207
16–18	16.4	1980.4±1.9	LD	LD	22.5	LD	49.4	14.2	86.1	9.9	132	30.2	42.2	214	LD	14.7	3.6	18.3	38.8	12.9	5.4	57.1	86.0	290	376
18–20	18.5	1976.6±1.9	LD	LD	20.5	LD	23.7	6.8	51.0	LD	90.7	LD	49.7	140	LD	LD	LD	LD	45.7	22.2	LD	67.9	51.1	208	259
20–22	20.6	1972.6±2.1	0.2	LD	35.1	LD	47.1	LD	82.2	LD	98.5	21.4	58.7	179	LD	10.5	51.9	62.4	39.8	12.4	2.5	54.7	82.4	296	378
22–24	22.8	1968.6±1.9	LD	LD	17.7	LD	74.4	1.0	93.1	6.3	12.7	15.5	25.6	60.1	LD	8.2	43.9	52.1	37.6	5.4	3.2	46.2	93.1	158	251
24–26	24.9	1964.6±2.0	LD	107	LD	1.3	6.7	21.8	137	26.7	34.4	150	LD	211	29.9	1.4	0.6	31.9	11.3	52.9	142	206	137	448	585
26–28	27.0	1960.8±1.9	1.3	LD	LD	3.6	LD	38.8	42.4	38.4	LD	75.6	3.9	118	13.9	0.9	19.5	34.3	LD	LD	109	109	43.7	261	305
28–30	29.1	1957.0±1.9	2.8	LD	51.7	LD	3.4	2.0	57.1	LD	2.4	LD	39.1	41.5	LD	0.6	36.6	37.2	2.0	LD	LD	2.0	59.8	80.7	140
30–32	31.3	1952.9±2.2	LD	LD	25.4	LD	7.4	LD	32.8	6.5	27.1	31.1	11.1	75.8	4.7	12.0	142	158	21.2	LD	7.3	28.5	32.8	263	295
32–34	33.7	1948.5±2.2	LD	LD	LD	4.1	LD	26.8	30.9	11.7	20.8	115	LD	148	0.3	LD	6.4	6.7	6.2	LD	52.5	58.7	30.9	213	244
34–36	36.1	1944.0±2.3	0.5	LD	93.6	LD	7.4	53.1	154	LD	22.4	32.1	56.8	111	LD	LD	64.6	64.6	LD	0.4	2.7	3.1	155	179	334
36–38	38.5	1939.7±2.0	LD	LD	LD	10.5	LD	21.4	31.9	6.9	13.4	34.9	LD	55.2	113	LD	17.6	130	2.8	LD	80.2	83.0	32.0	268	300
38–40	40.6	1935.8±1.9	LD	LD	LD	5.6	LD	21.3	26.9	LD	4.4	62.2	LD	66.6	1.2	25.2	33.3	59.7	LD	LD	98.0	98.0	26.9	224	251
40–42	42.8	1931.7±2.2	4.6	LD	61.0	19.9	2.0	LD	82.9	10.3	17.7	0.9	LD	28.9	LD	0.2	11.6	11.8	LD	21.0	LD	21.0	87.5	61.6	149
42–44	45.2	1927.2±2.3	LD	LD	93.3	LD	38.7	22.8	155	21.6	26.9	12.2	14.6	75.3	LD	LD	LD	LD	2.2	9.0	LD	11.2	155	86.5	241
44–46	47.6	1922.9±2.1	LD	LD	67.3	LD	LD	26.7	94.0	3.1	43.4	32.5	41.3	120	0.8	5.1	3.4	9.3	37.5	11.3	4.3	53.1	94.0	183	277
46–48	49.8	1918.7±2.1	LD	1.8	33.1	LD	42.9	88.9	167	11.2	12.6	26.9	18.2	68.9	4.2	LD	15.1	19.3	16.6	31.4	LD	48.0	167	136	303
48–50	52.2	1914.5±2.2	LD	LD	48.7	LD	48.7	LD	97.4	LD	39.3	LD	LD	39.3	LD	LD	LD	LD	6.1	LD	LD	6.1	97.4	45.4	143
50–52	54.5	1910.1±2.2	0.1	LD	64.2	28.1	4.8	LD	97.1	28.2	36.5	3.7	LD	68.4	LD	LD	16.7	16.7	9.9	18.4	0.9	29.2	97.2	114	211
52–54	57.0	1905.6±2.3	LD	LD	LD	0.6	LD	6.8	7.4	25.5	LD	11.6	LD	37.1	LD	7.1	172	179	LD	LD	LD	LD	7.4	216	224
54–56	59.4	1901.1±2.2	LD	0.5	36.7	LD	8.1	LD	45.3	LD	9.6	3.2	6.8	19.6	LD	LD	27.9	27.9	6.5	LD	LD	6.5	45.3	53.9	99.2
56–58	61.8	1896.6±2.2	LD	LD	29.2	LD	24.3	LD	53.5	LD	26.6	0.7	0.9	28.2	LD	LD	10.4	10.4	29.5	27.8	LD	57.3	53.5	95.9	149
58–60	63.9	1892.8±1.6	LD	LD	19.6	LD	6.5	LD	26.1	6.5	44.3	LD	LD	50.8	3.7	26.4	16.6	46.7	34.9	1.9	7.6	44.4	26.1	142	168
LD			1.64	1.11	1.20	1.85	2.28	3.70	-	1.79	1.95	2.40	6.41	-	5.21	4.82	5.23	-	3.75	5.02	5.37	-	-	-	-

0–60 cm（1892.8–2010.6): LPAHs range from 7.4 to 252 ng/g, with a mean value of 80.6 ng/g; HPAHs range from 45.4 to 477 ng/g, with a mean value of 223 ng/g; ∑PAHs range from 99.2 to 677 ng/g, with a mean value of 304 ng/g. Notes: "LD" represents below the limit of detection. M2, M3, M4, M5, and M6 represent the mean values of the 3-rings, 40-rings, 5-rings, and 6-rings, respectively. "-" represents that this value cannot be calculated mathematically.

Table S2. Molecular ratios of specific aromatic compounds in the marine sediment core from Daya Bay.

Molecular ratios	Phe/Ant	Ant/Ant+Phe	Fluo/Pyr	Fluo/Fluo+Pyr	BgP/InP	InP/InP+BaP	BaA/Chr	BaA/BaA+Chr
2009.0±1.6	0.78	0.56			2.52	0.28		
2005.5±1.8	1.19	0.46	0.04	0.04				
2002.0±1.7	0.02	0.98	0.77	0.43	4.25	0.19		
1998.6±1.8	0.76	0.57	0.18	0.15	0.35	0.74		
1995.2±1.6	39.6	0.02	0.03	0.03				
1991.7±1.8								
1988.0±1.9	0.65	0.60			10.7	0.09	68.1	0.99
1984.2±1.9					0.06	0.94	6.58	0.87
1980.4±1.9	3.48	0.22	0.08	0.07	0.14	0.88	0.72	0.42
1976.6±1.9	3.49	0.22						
1972.6±2.1					0.06	0.94	0.36	0.27
1968.6±1.9	74.4	0.01	0.50	0.33	0.09	0.92	0.61	0.38
1964.6±2.0	0.31	0.76	0.78	0.44	12.5	0.07		
1960.8±1.9							19.4	0.95
1957.0±1.9	1.70	0.37						
1952.9±2.2			0.24	0.19	0.34	0.74	2.80	0.74
1948.5±2.2			0.56	0.36	8.47	0.11		

1944.0±2.3	0.14	0.88					0.57	0.36
1939.7±2.0			0.51	0.34	28.6	0.03		
1935.8±1.9								
1931.7±2.2			0.58	0.37				
1927.2±2.3	1.70	0.37	0.80	0.45			0.84	0.46
1922.9±2.1			0.07	0.07	0.11	0.90	0.79	0.44
1918.7±2.1	0.48	0.67	0.89	0.47			1.48	0.60
1914.5±2.2								
1910.1±2.2			0.77	0.44	0.09	0.92		
1905.6±2.3								
1901.1±2.2							0.47	0.32
1896.6±2.2							0.78	0.44
1892.8±1.6			0.15	0.13	0.22	0.82		
Mean	9.19	0.48	0.43	0.27	4.57	0.57	7.96	0.55
Range	0.02-74.4	0.01-0.98	0.03-0.89	0.03-0.47	0.06-28.6	0.03-0.94	0.30-668.1	0.27-0.99

Table S3. Compound-specific $\delta^{13}C$ PDB(‰) isotopic compositions of PAHs in the marine sediment core from Daya Bay.

Calendar	2-rings	3-rings				4-rings				5-rings			6-rings			
	Nph	Acy	Acp	Flr	Phe	Ant	Fluo	Pyr	BaA	Chr	BbF	BkF	BaP	InP	DhA	Bgp
2009.0±1.6	LD	-25.28	-24.48	LD	-23.06	-18.87	-22.45	-30.29	-25.58	-21.22	-23.61	LD	-30.26	-21.91	-23.32	-24.05
2005.5±1.8	LD	-27.06	-17.74	-19.75	-25.71	LD	LD	-23.98	-26.80	LD	-20.97	LD	LD	-23.78	LD	-23.68
2002.0±1.7	LD	-20.71	-23.25	-24.80	-25.40	LD	-23.15	-22.01	-29.16	-22.16	-19.24	LD	-23.86	-29.78	-33.09	-28.25
1998.6±1.8	LD	LD	-19.56	LD	-27.08	-28.57	-24.21	-24.90	-25.01	-31.74	-25.36	-27.45	-28.72	-20.24	-23.38	-26.09
1995.2±1.6	LD	-20.53	LD	LD	-25.25	LD	-19.75	-26.82	-27.38	-23.07	-26.43	-30.87	-20.10	-25.05	-19.78	-20.18
1991.7±1.8	LD	-22.67	-20.45	-19.68	-17.08	LD	-21.58	-22.05	-30.67	LD	-20.63	-23.33	-22.06	-23.63	-26.00	-19.60
1988.0±1.9	LD	-21.49	LD	LD	LD	-20.32	-25.21	LD	LD	-20.22	LD	LD	LD	-19.90	-24.67	-19.85
1984.2±1.9	LD	-23.03	-17.09	-16.98	-25.00	LD	-24.26	-18.53	-22.75	-25.41	-27.84	LD	-31.95	-22.90	-21.28	-25.42
1980.4±1.9	LD	LD	LD	LD	-23.85	-21.89	LD	-23.82	-21.78	-19.77	-22.11	-20.38	-22.19	-20.58	-24.54	LD
1976.6±1.9	-18.87	-27.06	-21.80	LD	-17.45	-23.20	-23.27	-19.04	-25.69	-22.83	-22.07	-22.10	-22.56	-20.95	-22.41	-25.64
1972.6±2.1	LD	LD	LD	LD	LD	LD	LD	LD	LD	LD	LD	-24.86	LD	-26.28	-22.80	LD
1968.6±1.9	LD	-18.44	-25.11	LD	-25.33	-28.34	LD	LD	-21.49	-28.35	-28.78	-25.49	-24.49	LD	LD	-28.26
1964.6±2.0	LD	-24.95	LD	LD	LD	LD	LD	LD	-21.77	-27.45	-22.59	-25.89	LD	-22.32	-22.06	-24.21
1960.8±1.9	LD	LD	-21.16	-17.40	-20.99	-25.32	-18.32	-20.53	LD	LD	-19.90	-22.15	-24.87	-21.59	-17.63	-20.40
1957.0±1.9	-22.61	-18.91	-25.03	-23.26	-21.04	-25.15	-31.54	-18.22	-17.67	-19.62	-29.76	LD	-23.69	LD	-22.92	-21.41
1952.9±2.2	-27.43	LD	LD	LD	-21.63	LD	LD	-19.86	-32.24	LD	-30.40	LD	-19.50	-24.61	-26.43	-23.67
1948.5±2.2	LD	-17.83	LD	-28.56	-25.98	LD	-22.15	-19.40	LD	-21.04	-27.00	-29.65	-21.10	-17.32	-27.31	-17.72
1944.0±2.3	LD	-17.65	-24.13	-27.12	-22.22	LD	-20.05	-23.45	-21.16	-23.30	-25.34	-24.77	-24.19	-26.58	-17.20	-25.35
1939.7±2.0	LD	-26.32	LD	LD	-25.29	-25.77	-16.88	-22.94	-32.71	-24.23	-25.00	-29.72	-17.53	-18.63	-31.60	-23.19
1935.8±1.9	LD	LD	LD	LD	LD	LD	-22.60	-26.95	-25.20	-26.54	LD	LD	LD	LD	-27.53	-19.98
1931.7±2.2	LD	LD	LD	LD	-17.94	-16.72	LD	-16.68	-32.36	LD	-30.94	-22.67	-21.50	-24.14	-17.26	-20.06
1927.2±2.3	LD	-21.11	-24.63	-17.65	-20.68	-27.56	-16.84	-24.16	-26.08	-24.69	-26.32	-25.42	-23.41	-25.62	-18.76	-25.49
1922.9±2.1	-18.73	LD	-25.54	-17.59	-26.97	LD	-29.08	-30.21	-25.64	-22.19	-28.18	LD	-30.24	-25.00	-19.94	-25.95
1918.7±2.1	LD	-18.90	-17.29	LD	-24.02	-28.76	-28.37	-23.14	-22.21	-22.27	-22.19	-29.67	-27.78	-30.48	-27.50	-32.50
1914.5±2.2	-27.22	LD	-22.06	-17.13	-21.07	-28.19	-18.99	-24.78	-32.89	-24.41	-27.40	-27.67	-21.18	-26.98	-19.94	-22.91
1910.1±2.2	LD	LD	-21.45	-22.32	-23.57	-22.98	-22.24	-25.23	-32.82	-17.24	-23.61	-25.39	-21.66	-23.05	-21.27	-24.60
1905.6±2.3	LD	LD	LD	-28.58	-35.02	LD	-25.91	-31.61	-26.62	-20.69	-21.02	-30.46	-25.74	-23.39	-25.37	-27.25
1901.1±2.2	LD	LD	LD	LD	-21.45	-19.01	-20.28	-22.88	-30.04	LD	-28.98	-22.23	-19.19	-30.86	-26.62	-24.65
1896.6±2.2	LD	-19.32	-24.60	LD	-18.22	LD	-33.54	LD	LD	LD	-33.57	LD	LD	-19.92	-30.85	-27.58
1892.8±1.6	LD	LD	-24.43	LD	-16.14	LD	-34.18	LD	-30.91	-24.29	-26.17	-28.97	-21.57	-28.76	-21.51	-21.19
Mean	-22.97	-21.84	-22.21	-21.60	-22.98	-24.04	-23.69	-23.39	-26.67	-23.31	-25.39	-25.96	-23.72	-23.86	-23.68	-23.90

Note: "LD" represents below the limit of detection.

Table S4. Summary of δ^{13}C PDB(‰) isotopic compositions of PAHs in the marine sediment core from Daya Bay.

Calendar	2-rings	3-rings	4-rings	5-rings	6-rings	LPAHs	HPAHs	∑PAHs
2009.0±1.6	LD	−22.92	−24.89	−26.94	−23.09	−22.92	−24.74	−24.18
2005.5±1.8	LD	−22.57	−25.39	−20.97	−23.73	−22.57	−23.84	−23.27
2002.0±1.7	LD	−23.54	−24.12	−21.55	−30.37	−23.54	−25.63	−24.99
1998.6±1.8	LD	−25.07	−26.47	−27.18	−23.24	−25.07	−25.71	−25.56
1995.2±1.6	LD	−22.89	−24.26	−25.80	−21.67	−22.89	−23.94	−23.77
1991.7±1.8	LD	−19.97	−24.77	−22.01	−23.08	−19.97	−23.28	−22.26
1988.0±1.9	LD	−20.91	−22.72	LD	−21.47	−20.91	−21.97	−21.66
1984.2±1.9	LD	−20.53	−22.74	−29.90	−23.20	−20.53	−24.48	−23.27
1980.4±1.9	LD	−22.87	−21.79	−21.56	−22.56	−22.87	−21.90	−22.09
1976.6±1.9	−18.87	−22.38	−22.71	−22.24	−23.00	−21.68	−22.66	−22.33
1972.6±2.1	LD	LD	LD	−24.86	−24.54	LD	−24.65	−24.65
1968.6±1.9	LD	−24.31	−24.92	−26.25	−28.26	−24.31	−26.14	−25.41
1964.6±2.0	LD	−24.95	−24.61	−24.24	−22.86	−24.95	−23.76	−23.90
1960.8±1.9	LD	−21.22	−19.43	−22.31	−19.87	−21.22	−20.67	−20.86
1957.0±1.9	−22.61	−22.68	−21.76	−26.73	−22.17	−22.67	−23.10	−22.92
1952.9±2.2	−27.43	−21.63	−26.05	−24.95	−24.90	−24.53	−25.24	−25.09
1948.5±2.2	LD	−24.12	−20.86	−25.92	−20.78	−24.12	−22.52	−22.92
1944.0±2.3	−17.65	−22.78	−21.99	−24.77	−23.04	−22.78	−23.14	−23.04
1939.7±2.0	−26.32	−25.79	−24.19	−24.08	−24.47	−25.79	−24.24	−24.60
1935.8±1.9	LD	LD	−25.32	LD	−23.76	LD	−24.80	−24.80
1931.7±2.2	LD	−17.33	−24.52	−25.04	−20.49	−17.33	−23.20	−22.03
1927.2±2.3	−21.11	−22.33	−22.94	−25.05	−23.29	−22.33	−23.68	−23.23
1922.9±2.1	LD	−23.37	−26.78	−29.21	−23.63	−22.21	−26.27	−25.02
1918.7±2.1	−18.90	−22.24	−24.00	−26.55	−30.16	−22.24	−26.61	−25.36
1914.5±2.2	LD	−22.11	−25.27	−25.42	−23.28	−23.13	−24.72	−24.19
1910.1±2.2	LD	−22.58	−24.38	−23.55	−22.97	−22.58	−23.71	−23.39
1905.6±2.3	LD	−31.8	−26.21	−25.74	−25.34	−31.80	−25.81	−26.80
1901.1±2.2	LD	−20.23	−24.40	−23.47	−27.38	−20.23	−25.08	−24.20
1896.6±2.2	−19.32	−20.71	−33.54	−33.57	−26.12	−20.71	−29.09	−25.95
1892.8±1.6	LD	−20.29	−29.79	−25.57	−23.82	−20.29	−26.39	−25.28
Mean	−21.84	−22.65	−24.51	−25.19	−23.88	−22.61	−24.36	−23.90

Note: "-" represents below the limit of detection
